# Optimizing Trajectories for Cranial Laser Interstitial Thermal Therapy Using Computer-Assisted Planning: A Machine Learning Approach

**DOI:** 10.1007/s13311-018-00693-1

**Published:** 2018-12-05

**Authors:** Kuo Li, Vejay N. Vakharia, Rachel Sparks, Lucas G. S. França, Alejandro Granados, Andrew W. McEvoy, Anna Miserocchi, Maode Wang, Sebastien Ourselin, John S. Duncan

**Affiliations:** 1 The First Affiliated Hospital of Xi’an Jiaotong University, Xi’an, Shaanxi People’s Republic of China; 2 Department of Clinical and Experimental Epilepsy, UCL Institute of Neurology, 33 Queen Square, London, WC1E 6BT UK; 3 National Hospital for Neurology and Neurosurgery, Queen Square, London, UK; 4 Wellcome EPSRC Centre for Interventional and Surgical Sciences (WEISS), University College London, London, UK; 5 School of Biomedical Engineering and Imaging Sciences, St Thomas’ Hospital, King’s College London, London, UK

**Keywords:** Machine learning, LITT, MTLE, epilepsy, laser ablation.

## Abstract

Laser interstitial thermal therapy (LITT) is an alternative to open surgery for drug-resistant focal mesial temporal lobe epilepsy (MTLE). Studies suggest maximal ablation of the mesial hippocampal head and amygdalohippocampal complex (AHC) improves seizure freedom rates while better neuropsychological outcomes are associated with sparing of the parahippocampal gyrus (PHG). Optimal trajectories avoid sulci and CSF cavities and maximize distance from vasculature. Computer-assisted planning (CAP) improves these metrics, but the combination of entry and target zones has yet to be determined to maximize ablation of the AHC while sparing the PHG. We apply a machine learning approach to predict entry and target parameters and utilize these for CAP. Ten patients with hippocampal sclerosis were identified from a prospectively managed database. CAP LITT trajectories were generated using entry regions that include the inferior occipital, middle occipital, inferior temporal, and middle temporal gyri. Target points were varied by sequential AHC erosions and transformations of the centroid of the amygdala. A total of 7600 trajectories were generated, and ablation volumes of the AHC and PHG were calculated. Two machine learning approaches (random forest and linear regression) were investigated to predict composite ablation scores and determine entry and target point combinations that maximize ablation of the AHC while sparing the PHG. Random forest and linear regression predictions had a high correlation with the calculated values in the test set (*ρ* = 0.7) for both methods. Maximal composite ablation scores were associated with entry points around the junction of the inferior occipital, middle occipital, and middle temporal gyri. The optimal target point was the anteromesial amygdala. These parameters were then used with CAP to generate clinically feasible trajectories that optimize safety metrics. Machine learning techniques accurately predict composite ablation score. Prospective studies are required to determine if this improves seizure-free outcome while reducing neuropsychological morbidity following LITT for MTLE.

**Electronic supplementary material:**

The online version of this article (10.1007/s13311-018-00693-1) contains supplementary material, which is available to authorized users.

## Introduction

Surgery has been shown to provide long-term seizure remission in 2 thirds of patients with drug-resistant mesial temporal lobe epilepsy [1]. Hippocampal sclerosis (HS) is a common underlying cause, conventionally treated with anteromesial temporal resections whereby the temporal pole and variable amounts of the lateral neocortex are resected prior to an intraventricular amygdalohippocampectomy. Other approaches include transcortical, transsylvian [2], subtemporal [3], and selective amygdalohippocampectomy.

There has been recent interest in less invasive ablative techniques such as gamma knife [4], radiofrequency ablation [5], and laser interstitial thermal therapy (LITT) [6]. LITT has the advantage of being able to modulate the extent of the ablation cavity real-time through MR thermography and provides seizure freedom rates comparable to open surgery [6, 7] with potentially improved neuropsychological outcomes [8]. LITT has also been shown to be efficacious in mesial temporal lobe epilepsy (MTLE) without HS when the seizure onset zone has been confirmed with stereoelectroencephalography (SEEG) [9]. The extent of ablation of the mesial hippocampal head has been shown to be an independent predictor of seizure freedom [10], while sparing the parahippocampal gyrus (PHG), and surrounding structures may mitigate adverse effects on neuropsychological outcome [8, 11–13].

Selective amygdalohippocampal ablation with LITT is performed by stereotactically inserting a laser catheter along the longitudinal axis of the hippocampus to create an ablation diameter of 5 to 20 mm. The extent of ablation of the amygdalohippocampal complex (AHC), PHG, entorhinal cortex (EnC), and surrounding critical white matter fiber tracts is dictated by the planned trajectory. To aid LITT trajectory planning and improve ablation volumes, a systematic manual method utilizing the “posterior-inferior corridor” has been proposed [14]. Comparisons of manually planned with computer-assisted planning (CAP) of trajectories showed that the latter improved AHC ablation, reduced the unablated mesial hippocampal head remnant, spared the PHG, and enhanced safety metrics [15]. CAP also provides a uniform objective method of trajectory planning that could help to overcome the initial learning curve associated with implementing a novel technology.

 Here, we aim to implement machine learning techniques with CAP to optimize parameters for LITT that will maximize AHC ablation, sparing of PHG, and distance from the brainstem, sulci, and vasculature.

## Methodology

### Subjects

**Fig. 1:**
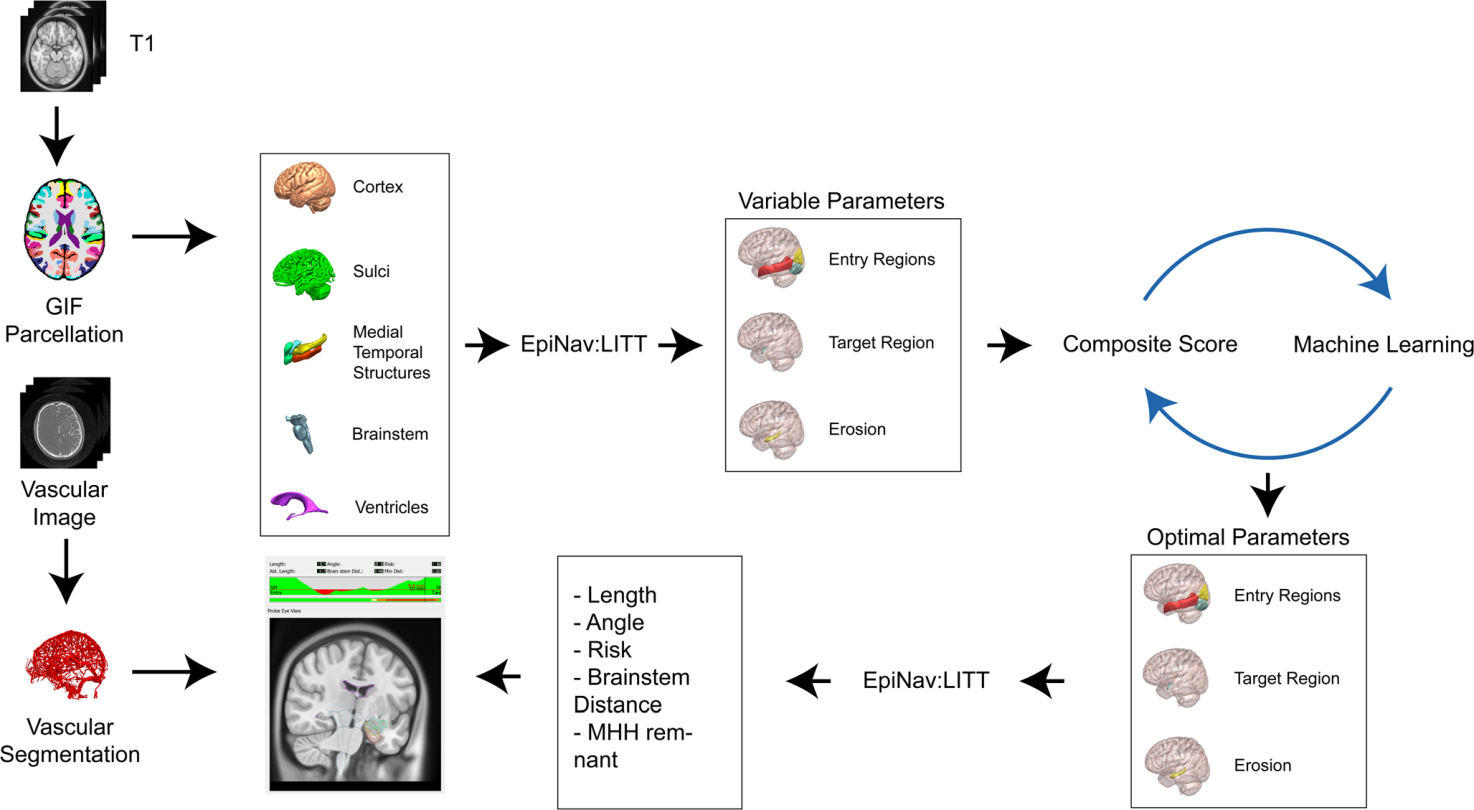
Study flowchart. Trajectory planning requires a single T1 image from which a whole-brain parcellation and pCT (not shown) are generated using geodesic information flows (GIF). Important structures required for planning were automatically segmented from the GIF parcellation, and 3D models were generated of the cerebral cortex, sulci, mesial temporal lobe structures (hippocampus, amygdala, entorhinal cortex, parahippocampal gyrus), brainstem, and ventricles. The automated trajectories were then calculated using EpiNav based on all possible combinations of entry region, target region, and AHC erosion. Composite scores for each of the trajectories were calculated. Machine learning was performed on 50% (training set), and composite score predictions were compared with the actual calculated values in the remaining 50% (test set). Parameters for entry region, target region, and erosion were defined. In combination with patient-specific vascular segmentations, the machine learning parameters were then used on a patient-specific basis for computer-assisted planning (CAP) to optimize safety metrics such as laser catheter intracerebral length, drilling angle to the skull, risk score, and distance from the brainstem and mesial hippocampal head (MHH) remnant following simulated ablation (applying a 15 mm ablation diameter) based on user-specified parameters

Ten consecutive preoperative patients with HS (5 left) that were eligible for LITT were identified from a prospectively maintained database at the National Hospital for Neurology and Neurosurgery, London. From a single T1 MPRAGE acquisition with a voxel size of 1 mm isotropic (TE/TR/TI = 3.1/7.4/400 ms; flip angle 11°; parallel imaging acceleration factor 2) whole-brain parcellations and synthetic CT (pseudo-CT), images were generated using geodesic information flow [16] and a multi-atlas information propagation scheme [17], respectively. Patient-specific 3D models of the cortex, ventricular system, brainstem, AHC, PHG, EnC, and sulci were extracted from the whole-brain parcellation (see Fig. 1). EpiNav™ (UCL, CMIC) was then employed as CAP to generate trajectories based on a previously described algorithm [15].

### Computer-Assisted Planning

In brief, the algorithm aims to minimize the intracerebral catheter length; drilling angle from orthogonal to skull and PHG ablation, while maximizing distance from critical structures (sulci and intracranial vasculature); ablation of the mesial hippocampal head and AHC; and distance from the brainstem (see Fig. 1). First, trajectories that do not meet the hard constraints of angle < 35°, length < 120 mm, distance to brainstem > 7.5 mm, and ventricle avoidance are rejected. Next, distance to critical structures is optimized where the minimum distance was set to 3 mm (*user-defined constraint*) [18]. Overall risk score was calculated based on a cumulative distance from critical structures along the entire length of the trajectory and normalized between 0 and 2 [19]

$$ R=\Big\{{\displaystyle \begin{array}{c}\sum \limits_i^N\frac{10-\mathrm{Dist}(i)}{N\left(10-3\right)},\mathrm{Dist}(i)>3\\ {}1+\sum \limits_i^N\frac{3-\mathrm{Dist}(i)}{3N},\mathrm{Dist}(i)\le 3\end{array}}\operatorname{} $$

where *i* refers to the indices of nodes along the trajectory (a total of *N* = 128 nodes) where distance measures to the closest critical structure are performed. Distances from a critical structure at any given node (*i*) that are > 3 mm are calculated by the top row of the equation, while distances < 3 mm by the bottom row. A risk of 0 therefore indicates that the entire trajectory remained > 10 mm from a critical structure along its entire length, while a score of 1 indicates a constant distance of 3 mm. Scores > 1 are attributed to trajectories that pass less than 3 mm from a critical structure and would therefore carry a theoretically greater risk of hemorrhage.

To optimize trajectory parameters, CAP trajectories were calculated for all possible combinations of the following parameters for each patient:

1. Entry zones (inferior occipital gyrus (IOG), middle occipital gyrus (MOG), inferior temporal gyrus (ITG), and middle temporal gyrus (MTG)
2. Morphological erosions (circumferential diminution) of the AHC (0 mm, 1 mm, and 2 mm)
3. Target zones (translations of the centroid of the amygdala by 0 to 3 mm in the *X*, *Y*, and *Z* planes)

Based on these parameter combinations, a total of 760 trajectories per patient were generated using CAP and a composite score was calculated by the following equation:

$$ \mathrm{Composite}\ \mathrm{score}\ \left(\mathrm{ml}\right)=\frac{\mathrm{AH}{\mathrm{C}}_{\mathrm{ablation}}}{\mathrm{AH}{\mathrm{C}}_{\mathrm{anatomical}\ \mathrm{volume}}}-\frac{\mathrm{PH}{\mathrm{C}}_{\mathrm{ablation}}}{\mathrm{PH}{\mathrm{C}}_{\mathrm{anatomical}\ \mathrm{volume}}} $$

where AHC and PHC indicate the volumes (ml) of the correspondent structures.

Ablation volumes were calculated based on a conservative estimate of maximal ablation diameter of 15 mm, which we have previously shown as an accurate reflection of the postablation cavity [15]. The planning system subsequently guides the surgeon as to the length of the laser ablation required to achieve the predicted ablation volumes. During the intervention, under MR thermography guidance, the surgeon is then able to iteratively withdraw the laser catheter to achieve a contiguous ablation cavity.

### Machine Learning

Two different supervised regression-based machine learning models (random forest and linear regression) were configured using R [20] and auxiliary packages: ggplot2 [21], randomForest [22], stringr [23], cowplot [24], RColorBrewer [25], and reshape2 [26].

All composite scores were normalized using the maximum score in each patient, in order to guarantee the comparability of these indices. The data was then split into training and testing sets. The training set of 5 patients (3800 trajectories) was selected at random. The data from the remaining 5 patients (3800 trajectories) were then used as the test dataset to validate the predictive accuracy of the model.

Both linear regression and random forest [27] models were trained on the first portion of the data, according to the parameters’ entry area (MTG, MOG, IOG, ITG), translations of the centroid of the amygdala (axial, sagittal, and coronal planes), and the magnitude of erosion of the AHC. For the random forest method, 100 trees were used. Linear regression was performed according to the following equation:

$$ \mathrm{Score}=\sum \limits_i{C}_i{V}_i $$

where *C* is the coefficient and *V* is the variable. The index *i* identifies the different variables/coefficient shown in Fig. 3.

We have then applied the trained predictors to the (unseen) test data and evaluated the obtained results. Pearson correlations between the predicted and actual composite score values were obtained, as well as the root mean square of the errors. The most important parameters to maximize the composite score were then evaluated.

Ethical approval was sought from the National Research Ethics Service Committee London, with approval reference 12/LO/0377. Individual patient consent was sought for use of anonymized perioperative imaging.

## Results

**Fig. 2:**
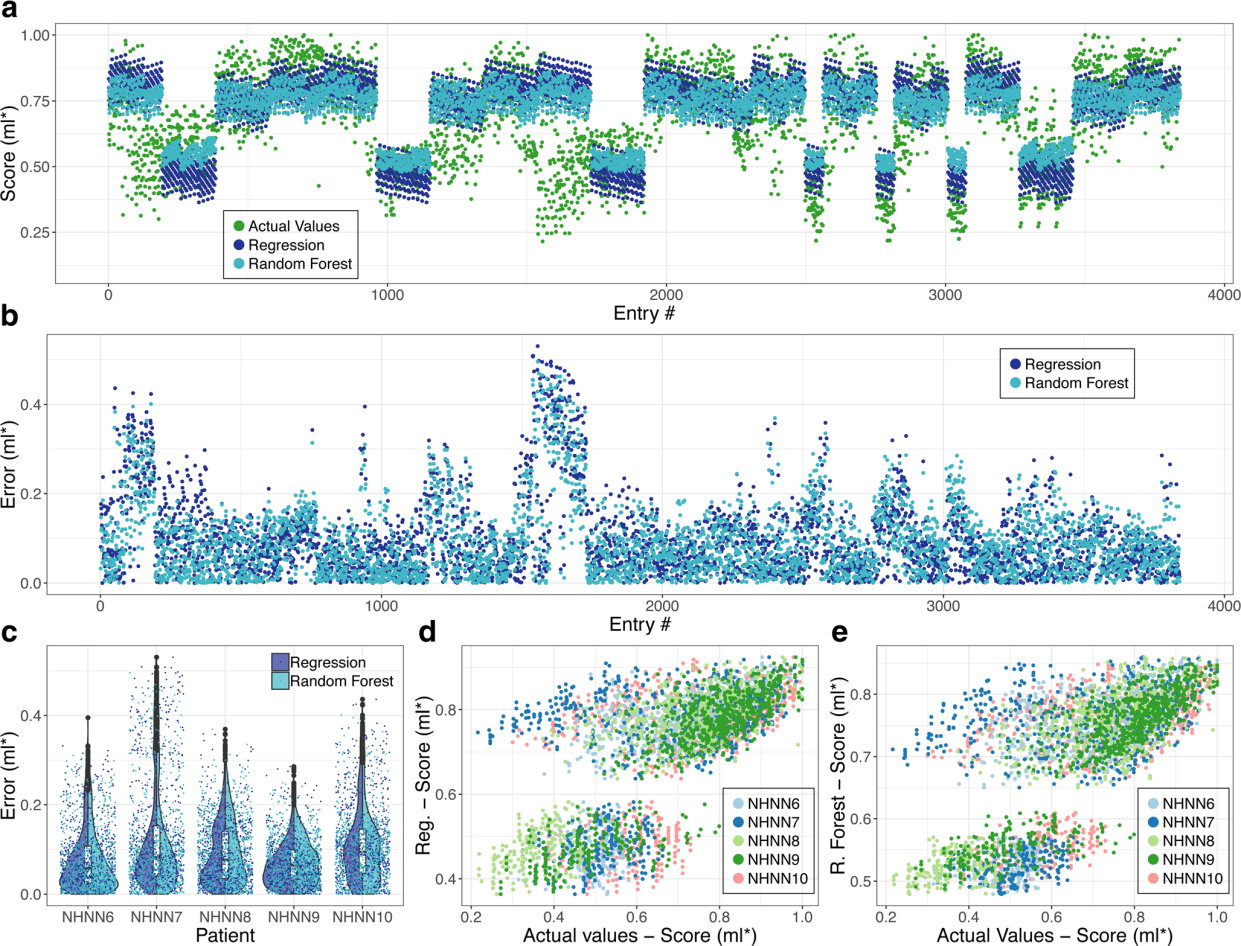
Evaluation of the accuracy of trained models (linear regression and random forest). (**A**) Comparison between values predicted by both models and composite score for all entries in every patient of the test set. (**B**) Error for both models, i.e., the difference between predicted and calculated composite scores, for all entries in every patient of the test set. (**C**) Violin plots of distributions errors shown in (**B**). (**D**) Scatter plot of composite scores versus predicted score (linear regression) for all entries in every patient of the testing set; each patient is represented by a different color. (**E**) Scatter plot of composite versus predicted scores (random forest) for all entries in every patient of the test set; each patient is represented by a different color. Both models were capable of reproducing the overall trend of data with good accuracy, with a Pearson correlation of *ρ* = 0.7 for the linear regression model and *ρ* = 0.7 for the random forest model. Some of the patients exhibit worse fitting and higher errors, e.g., NHNN7 and NHNN10. *ml = milliliters (normalized)

Both linear regression and random forest approaches showed similar results. The predicted scores reproduce the structure with good accuracy as shown by a Pearson correlation of *ρ* = 0.7 for both methods suggesting a strong correlation. Linear regression and random forest models featured a root-mean-square error (RMSE) of 0.13 and 0.12, respectively. Figure 2 shows the predicted values and actual scores.

**Fig. 3:**
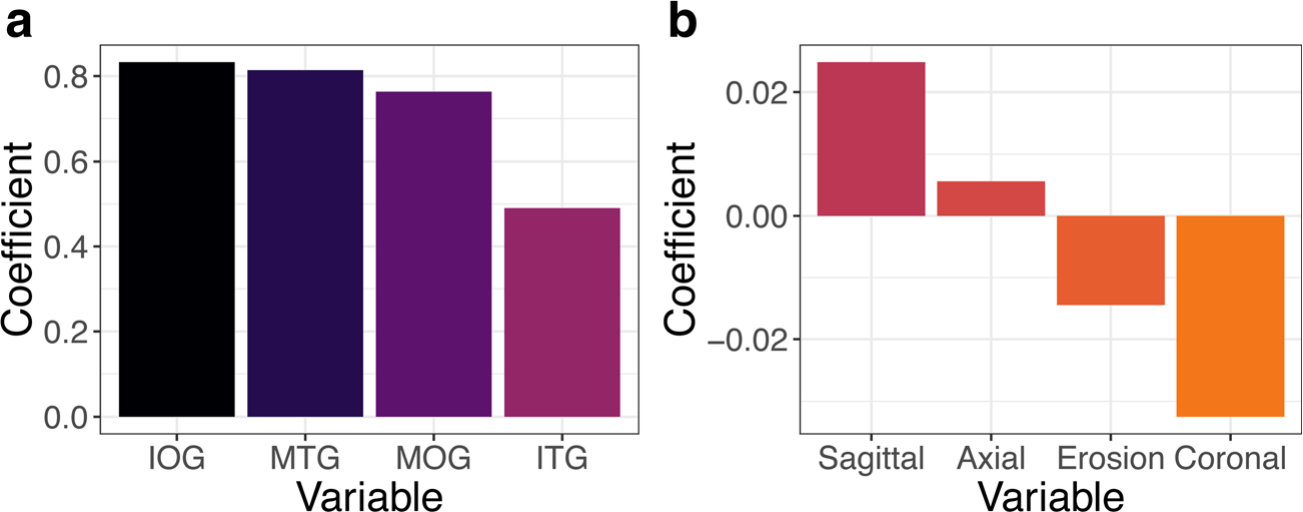
Coefficient *C*_*i*_ for each variable *V*_*i*_ of the estimated linear model. (**A**) Coefficients for entry areas. (**B**) Coefficients for target areas (translations of the centroid of the amygdala) and erosion of the AHC. Coronal shifts and erosion of the AHC impact negatively on the predicted scores. Note that coefficients for entry points are an order of magnitude greater than those for target points

**Fig. 4:**
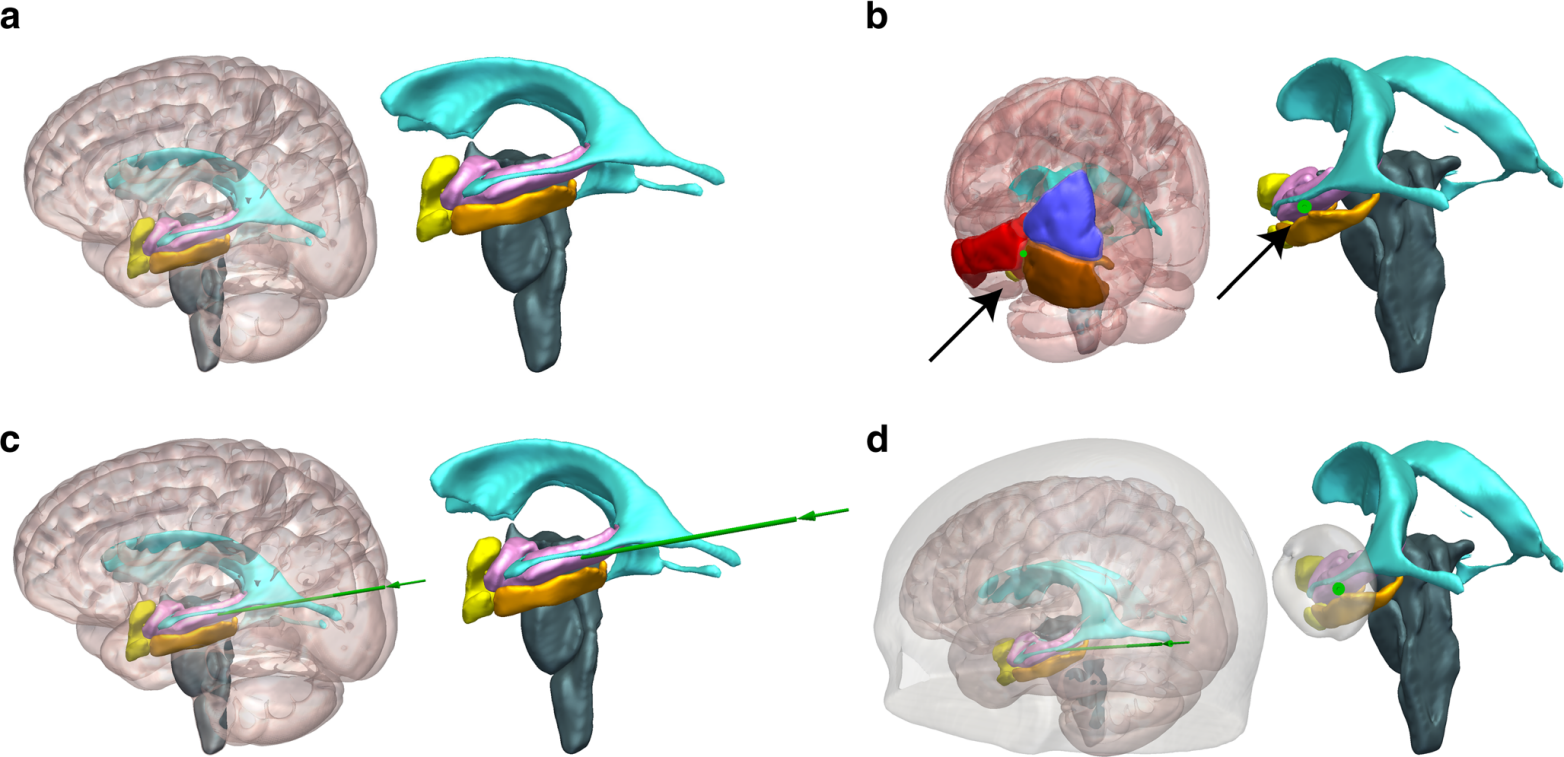
Anatomical structures and example LITT trajectory for amygdalohippocampectomy, shown with and without transparent cortical overlay. (**A**) Automated segmentations of patient-specific anatomical structures including the lateral ventricles (cyan), amygdalohippocampal complex (pink), entorhinal cortex (yellow), parahippocampal gyrus (orange), and brainstem (teal). (**B**) Probe’s eye view along trajectory (green), shown by implementing entry (black arrow) through the junction of middle occipital gyrus (yellow), inferior occipital gyrus (pink), and middle temporal gyrus (orange). (**C**) Lateral view of trajectory shown in (**B**), revealing entry into the hippocampus at the level of the tectum without crossing the occipital horn of the lateral ventricle. (**D**) Automated LITT trajectory with overlying transparent scalp and cortex. Right panel shows an entry zone at the level of the cortex based on the results of the linear regression

When identifying the most important component features, both linear regression and random forest methods also provide similar results. Figure 3 shows the coefficients for each variable of the linear regression. According to the values, in order to maximize the ablation score, one should prioritize an entry which is centered at the junction of the IOG, MTG, and MOG (see Fig. 4B, D) and apply an anterior and mesial translation to the centroid of the amygdala. Figure 4 shows an example of CAP trajectory generated using the machine learning parameters derived from the linear regression.

## Discussion

Here, we utilize machine learning algorithms to define the junction between the IOG, MOG, and MTG as the entry and anterior and medial translation of the amygdala as the target point parameters for LITT trajectories. Application of these parameters results in maximal ablation of the amygdalohippocampal complex and sparing of the parahippocampal gyrus. Prospective studies are now required to determine if this approach results in improved seizure-free outcome and reduced neuropsychological morbidity.

### Laser Interstitial Thermal Therapy

LITT for MTLE is a novel minimally invasive approach for selective amygdalohippocampectomy [28]. As with all novel therapies, there is an initially learning curve and early outcome results can be variable. To date, most case series of LITT for MTLE are limited in number (< 30), provide short follow-up durations of 6 to 24 months, and lack comprehensive reporting of secondary outcomes, such as neuropsychological and neurological morbidity.

Only a single “open-label” comparative study has been reported to date in which LITT was compared to open microsurgery [8]. This study included 39 patients that had undergone open microsurgery composed of tailored resection (18/39), standard ATL (4/39), and transcortical selective amygdalohippocampectomy (17/9) compared to 19 patients with LITT. At 6-month follow-up Engel 1 outcome was 61.5% and 57.9% for open surgery and LITT, respectively. Patients undergoing open surgery had a significant decline in naming or recognition tasks in > 80% (32/39) while this was not observed in any patients following LITT. It was suggested that this function may be subserved by the parahippocampal gyrus and/or surrounding white matter fiber tracts running within the temporal stem (uncinate fasciculus and inferior fronto-occipital fasciculus) or basal temporal regions (inferior longitudinal fasciculus) that are relatively spared following LITT.

A further study correlated implemented LITT trajectories with seizure outcome at a mean follow-up duration of 22.4 months in 23 patients [10]. Seizure-free (defined as Engel 1) outcomes across the whole cohort were 65% which rose to 73% when only considering patients with MTS. This study found that trajectories passing more mesially within the hippocampal head were more likely to be associated with seizure-free outcome. Quantitative analysis of the mesial hippocampal head remnant found patients who were Engel 1 had a remnant depth of 1.3 ± 0.5 mm compared to Engel 2 to Engel 4 of 3.7 ± 1.1 mm. To date, this remains the only significant independent predictor of seizure-free outcome that can be directly influenced by preoperative trajectory planning.

The largest single series to date reports outcomes from 58 patients in which 53.4% achieved Engel 1 outcome at a > 12-month mean follow-up while a similar series of 43 patients reported 67.4% Engel 1 outcome at a > 12-month mean follow-up. In both series, no significant difference in seizure-free outcome was found with mean percentage ablation volume of any structure. Of interest, Gross et al. [6] report that in 3 cases that did not initially achieve seizure-free outcome, a repeat ablation of residual medial temporal tissue did result in Engel 1 outcome. This supports the finding of Jermakowicz et al. regarding ablation of the mesial hippocampal head [10]. The difference in outcomes may, in part, be due to a lack of consistency between surgeons when planning trajectories, which automated systems can control for.

Visual field deficits are the most common neurological morbidity following LITT with reports ranging from 5 to 29% [7, 29, 30]. Contralateral visual field deficits can arise from damage to the optic radiation posteriorly or the lateral geniculate nucleus (LGN). Damage to the optic radiation would be expected to result in varying degrees of superior homonymous quadrantanopia while damage to the LGN would result in a complete homonymous hemianopia. Damage to the optic radiation is more likely with ablation cavities that extend into the sagittal striatum (periventricular white matter lateral to the posterior hippocampus). The LGN lies in the dorsal thalamus, occupying a medial position in the temporal horn of the lateral ventricle, and is, therefore, particularly susceptible to thermal injury during LITT at the level of the body of the hippocampus. Trajectories that pass superiorly within the hippocampus or in patients where the CSF/choroid plexus volume is particularly low have been suggested to carry a greater risk of LGN injury [30]. EpiNav-generated–optimized trajectories maximize distance from critical structures, including the brainstem, which, in our automated segmentation, incorporates the LGN.

### Computer-Assisted Planning

CAP is the use of computer algorithms to aid in preoperative surgical planning and simulation. CAP has been utilized in neurosurgical stereotaxy for over 3 decades. Initially, CAP was utilized by Davis et al. [31] to automate the calculation of stereotactic frame coordinates for manually chosen brain biopsy targets. Incremental improvements in CAP systems, mainly for stereotactic lesioning and deep brain stimulation, saw the introduction of multiple (generic) brain atlases that could be registered to patient MRI images to aid with precise target selection. The NeuroPlanner software incorporated 2 commonly used 3D stereotactic atlases to improve functional outcome following pallidotomy and pallidal DBS [32]. Using this platform, the surgeon was able to select the desired entry and target points in ~ 60 s and overall planning time was reduced to ~ 5 min. As computer processing power has increased multimodal image registration [33], automated segmentation [34] and 3D rendering [35] have allowed neurosurgical planning to become quicker and more sophisticated.

Software such as EpiNav allows the calculation of hundreds of thousands of possible trajectory permutations returning those that most closely fit the user-defined trajectory parameters [19]. Initial versions required the surgeon to precisely place target points, and the algorithm would return the trajectory with the lowest calculated risk, shortest intracerebral length, orthogonal drilling angle, and maximal gray matter sampling [36]. Later versions have incorporated whole-brain parcellations to allow the software to define the safest entry and target point combinations within user-defined anatomical structures [37]. CAP for SEEG follows similar algorithms in which electrode length, drilling angle to the skull, and gray matter sampling are optimized, while maximizing distance from critical structures such as vasculature. SEEG, however, has the added the complexity of multiple trajectories, and as such, calculations for optimal combinations of trajectories are required that provide adequate sampling of target structures while preventing electrode conflicts with other electrodes or blood vessels [19, 38]. Comparative studies between CAP and manually generated trajectories for SEEG have shown that SEEG plans can be generated with improved safety metrics in a fraction of the time needed for manual planning [34, 37], with no reported differences in feasibility from blinded external reviewers [39].

**Fig. 5:**
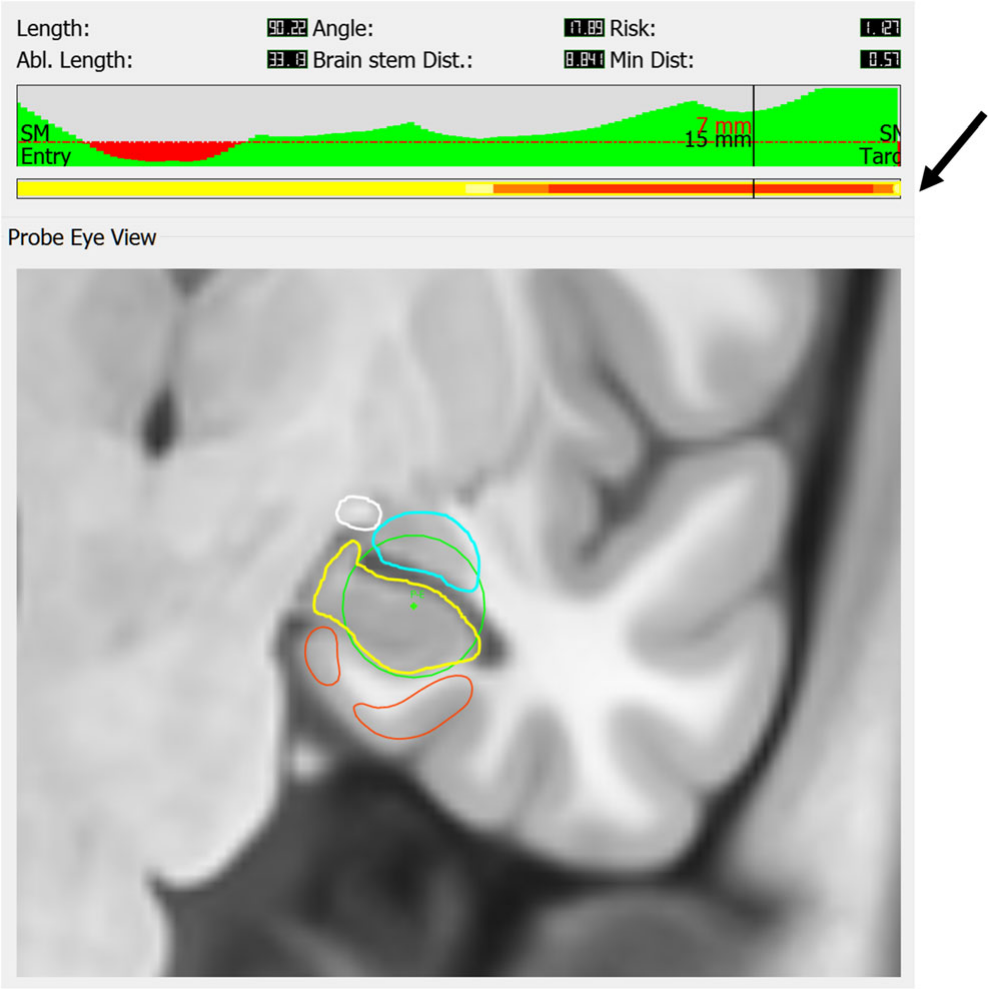
An example of a left-sided ablation with the “probe’s eye view” at the level of the head of the hippocampus. The parameters provided to the surgeon (top panel) are catheter length (mm), drilling angle to the skull (degrees), risk score, ablation length (mm), brainstem distance (mm), and minimum distance from critical structure (mm). A graphical depiction of the distance from vasculature along the length of the trajectory (green trace) is also provided. Distances to critical structures (sulci and vasculature) below the user-specified 3 mm (dashed red line) are shown as red regions. A numeric readout is provided at the position along the trajectory; e.g., at 15 mm from the target, the distance from a critical structure is 7 mm. The next panel (black arrow) is a color bar guiding the surgeon regarding the expected ablation length (red) upon which the volume calculations are base. The orange section depicts the region around the posterior hippocampus where continued ablation would result in additional hippocampal ablation, but at the expense of collateral structures. This yellow section depicts where continued ablation would not result in hippocampal ablation. Structures segmented are amygdala (blue), hippocampus (yellow), optic tract (white), and parahippocampal gyrus (orange). The laser position is shown as a green dot and the 15-mm ablation cavity as a green circle. Please note the distance calculations take into account the radius of the catheter and are not from the center of the trajectory

We previously applied CAP to LITT amygdalohippocampal ablation and independently generated trajectories in 25 patients [15]. Using parameters derived from expert opinion, the entry point was restricted to the inferior occipital gyrus. The target point of the centroid of the amygdala was translated by 3 mm anteriorly, mesially, and inferiorly to improve ablation of the mesial hippocampal head, maximize amygdala and entorhinal cortex ablation, and prevent heat dissipation to the pallidum, respectively. EpiNav^ TM^ provides guidance to the surgeon regarding trajectory safety parameters, length of laser catheter pull backs and anatomical ROI overlap (see Fig. 5).

As conventional in LITT trajectory planning, an extraventricular approach was implemented, entering the hippocampus at the level of the tectum and maximizing distance from the brainstem to prevent inadvertent thermal injury [14]. Due to individual patient anatomical variability, an entry point through the inferior occipital gyrus was only possible in 72% (18/25) of cases with the remainder passing through the middle occipital and middle temporal gyri. Ablation volumes with CAP were significantly improved by 11% for the AHC while the unablated mesial hippocampal head and PHG volume were reduced by 73% and 11%, respectively [15]. In the current study, we have implemented machine learning to determine the trajectory parameters that maximize the composite score. Through the calculation of 760 trajectories per patient across 10 patients, we computed all possible trajectory combinations and utilized machine learning to predict a normalized composite score of ablation volumes.

We previously applied target translations to the amygdala of ± 3, − 3, and − 3 for the axial, sagittal, and coronal planes, respectively. Our results suggest that the application of the machine learning parameters would increase the composite ablation scores achieved in our previous study by a further 10%, based on the refined entry point and if the coronal translation and AHC erosion are not applied.

In addition to optimizing trajectory parameters, CAP algorithms provide an objective means of generating trajectories that overcomes the heterogeneity in planning between different surgeons and institutions. As further knowledge regarding important surgical parameters is elucidated, these can be used to update the algorithm in an evidence-based systematic fashion. Prospective clinical studies are required to determine if LITT trajectories generated using these parameters will result in improved seizure-free outcome rates and reduced neuropsychological morbidity.

### Machine Learning Results

In order to identify the parameters for LITT amygdalohippocampal ablation that maximize the ablation composite score, we trained both linear regression and random forest machine learning models. To prevent overfitting, the models were trained on half of the dataset and then validated on the remaining unseen data. Both linear regression and random forest methods show similar outputs when compared to the actual scores, indicating a high reliability for the machine learning.

The results showed good correlation with the composite scores and low root-mean-square error, suggesting a good predictive accuracy. From all the parameters, the entry area had the most relevant impact on composite score with IOG, MTG, and MOG having the greatest contribution. These findings are consistent with the postero-inferior corridor described by Wu et al. [14], which is the only study in the literature that has been able to systematize manual LITT trajectory planning to improve ablation volume.

With regard to the target regions, translation of the centroid of the amygdala anteriorly (sagittal plane) and mesially (axial plane) resulted in the greatest increase in composite score while erosion of the AHC and inferior translation (coronal plane) had a negative impact. This would be expected as inferior trajectories ablate more of the PHG and spare the superior amygdala.

### Limitations

Only 10 patients, from a single center, were included in this study. The large number of possible unique trajectory combinations resulted in 7600 CAP-generated trajectories for evaluation. Future studies using larger datasets from a range of institutions and a number of neurosurgeons performing manual planning will be required to assess the external validity and robustness of CAP. Furthermore, the application of the linear regression results from this study will allow a more focused investigation of trajectory parameter combinations, therefore reducing the number of CAP-generated trajectories per patient.

A composite score was devised for the purposes of the machine learning. This is a pragmatic score that was devised due to a lack of definitive evidence in the literature. By maximizing the ablation of the AHC, we aim to ensure that the laser trajectory achieves the goal of the intervention, which is to ablate the sclerotic mesial temporal structures, with minimum collateral damage. Our previous work has also shown that this also significantly reduces the extent of the mesial hippocampal head remnant by 73% when compared to expert manually planned trajectories [15], which, to date, is the only independent predictor of seizure freedom [10]. In addition, although there is no definitive evidence that damage to the PHG alone is responsible for memory impairment, animal studies have shown that this induces severe memory impairment despite sparing the AHC. Trajectories sparing the PHG are also likely to spare adjacent structures such as the inferior longitudinal fasciculus that may have an important role in this function.

All imaging was derived from a single center, and the acquisition quality of the T1 image has significant implications for the whole-brain parcellation. It is unclear whether T1 acquisitions with movement artefact or sequences with poorer gray–white matter differentiation would result in less accurate structure segmentation and alter the CAP trajectory output.

The GIF whole-brain parcellation was derived from a control dataset. The resulting segmentation of the hippocampus overestimated hippocampal volume in our cohort of HS patients by ~ 12% [15]. A whole-brain parcellation derived from patients with HS may therefore provide more accurate hippocampal segmentations. Given that the linear regression results revealed a negligible effect of AHC erosion on the composite score, however, this is unlikely to have a significant effect on the result.

To calculate ablation volumes of anatomical structures, we have applied a uniform 15 mm ablation diameter to simulate the ablation cavity. We have shown in our previous work that across the 25 patients in that study, the application of a 15 mm ablation diameter resulted in a mean overestimate of the AHC ablation by + 4.96% [15]. Due to the complexities of modelling asymmetric ablation cavities as a result of variable heat dissipation from differential CSF flow, and the lack of a validated method to do this, we opted to apply the uniform 15 mm ablation diameter in these cases.

## Conclusion

To our knowledge, this is the first direct clinical application of machine learning for preoperative planning in stereotactic neurosurgical procedures. LITT is a novel therapy, and given the potential neuropsychological benefits with comparable seizure freedom rates, it is likely to be a viable alternative to hippocampal resection in the near future, as it spares the temporal neocortex, requires a shorter hospital stay, reduces a risk of complications, and promotes quicker recovery. As more neurosurgical units adopt this technology, it is vital that prior experience from high-volume centers is integrated within the decision making and preoperative planning process to minimize the learning effect on patients. Future work should concentrate on identifying and utilizing CAP to preserve important white fiber tracts that subserve distinct neuropsychological functions.

The use of machine learning in this context has allowed quantification of hitherto unidentified trajectory parameter combinations to be determined. When used with CAP, this allows contemporary research findings to be applied systematically and objectively across all centers. A prospective clinical trial implementing these machine learning parameters is required to determine if this translates into improved seizure-free outcomes and reduced neuropsychological morbidity.

## Electronic supplementary material

ESM 1 (PDF 1224 kb)
